# Vitamin D Levels as a Marker of Severe SARS-CoV-2 Infection

**DOI:** 10.3390/life14020210

**Published:** 2024-01-31

**Authors:** Lambros Athanassiou, Ifigenia Kostoglou-Athanassiou, Sofia Nikolakopoulou, Alexandra Konstantinou, Olga Mascha, Evangelos Siarkos, Charilaos Samaras, Panagiotis Athanassiou, Yehuda Shoenfeld

**Affiliations:** 1 COVID-19 Department, Asclepeion Hospital, Voula, GR16673 Athens, Greece; lambros.ath@gmail.com (L.A.); sofnikol@yahoo.gr (S.N.); alexkonstantinou2@gmail.com (A.K.); esiarkos@gmail.com (E.S.); xarisamar@gmail.com (C.S.); 2 Department of Endocrinology, Asclepeion Hospital, Voula, GR16673 Athens, Greece; 3 Department of Biochemistry, Asclepeion Hospital, Voula, GR16673 Athens, Greece; olmascha@gmail.com; 4 Department of Rheumatology, St. Paul’s Hospital, GR55134 Thessaloniki, Greece; pathanassiou@yahoo.gr; 5 Zabludowicz Center for Autoimmune Diseases, Sheba Medical Center, Reichman University, Herzelya 4610101, Israel; yehuda.shoenfeld@sheba.health.gov.il

**Keywords:** vitamin D, SARS-CoV-2, outcome, ferritin, d-dimers, fibrinogen

## Abstract

The SARS-CoV-2 virus may cause severe infection, which is associated with diverse clinical manifestations. Vitamin D has immunomodulating properties and may enhance the body’s defense system against invading pathogenic organisms. The aim was to assess 25(OH)D_3_ levels in patients hospitalized for severe infection from the SARS-CoV-2 virus and explore the relationship between 25(OH)D_3_ and outcomes. In a group of 88 patients hospitalized for severe infection from the SARS-CoV-2 virus and a control group matched for age and sex, the levels of 25(OH)D_3_ were analyzed. Levels of 25(OH)D_3_ were 17.36 ± 8.80 ng/mL (mean ± SD) compared with 24.34 ± 10.34 ng/mL in patients with severe SARS-CoV-2 infection and the control group, respectively, *p* < 0.001 (Student’s *t*-test). 25(OH)D_3_ levels were significantly related to outcomes, i.e., survival as opposed to non-survival, as more patients with 25(OH)D_3_ deficiency (0–10 ng/mL) and insufficiency (10–20 ng/mL) had a fatal outcome as compared with those with vitamin D sufficiency (*p* < 0.001, chi-square test, *p* < 0.001, Fisher’s exact test). Levels of 25(OH)D3 were inversely related to C-reactive protein (CRP), ferritin, d-dimer, and fibrinogen levels (*p* < 0.001, linear regression analysis, beta coefficient of variation, −0.176, −0.160, −0.178, and −0.158, respectively). Vitamin D deficiency observed in severe SARS-CoV-2 infection was related to disease outcomes.

## 1. Introduction

The SARS-CoV-2 virus was the initiating agent of the recent pandemic, likely having originated in the Wuhan region of China [1,2]. The pandemic surprised the medical profession and society, and no medical treatment was known at the time for the novel viral infection. Therefore, intense research was initiated to combat and prevent the disease, leading to the discovery of various agents capable of fighting the infection [3]. In particular, research into the matter of inadequate vitamin D, which may be related to severe infection and adverse outcomes, led to the observation that severe COVID-19 infection may be related to inadequate vitamin D levels [4,5,6,7]. Vitamin D is a hormone related to calcium and bone metabolism with significant immune-modulating properties [8]. In particular, it has been shown that vitamin D may act to enhance the innate immune response [9] and contribute to the response of the organism to infectious microorganisms, such as mycobacterium tuberculosis [10] and mycobacterium leprae [11]. Vitamin D induces the production of cathelicidin, thus having an antiviral effect [12]. However, vitamin D has multiple effects on the immune system. In particular, it may modulate the immune response. Daneshkhah et al. [13] investigated the possible effect of vitamin D deficiency on the development of cytokine storm and subsequent mortality in COVID-19 patients, and they concluded that the possible role of vitamin D in modulating the rate of cytokine storm in COVID-19 should be further investigated. The overproduction of inflammatory cytokines during severe COVID-19 disease is involved in the pathogenesis of the cytokine storm and may lead to mortality [14]. Vitamin D, via its anti-inflammatory properties [15], may modulate the cytokine storm observed in severe COVID-19 disease [16]. Consequently, vitamin D administration was implemented in the prevention and treatment of SARS-CoV-2 infection [17,18,19].

Dexamethasone was applied as first- and second-line treatments and led to the discovery that dexamethasone may prevent severe disease and improve adverse outcomes in the form of hospitalization within the acute care unit, as well as intubation [20,21]. Infection from the SARS-CoV-2 virus may manifest as a mild viral illness or may take the form of a severe disease, which may lead to adverse outcomes [22]. In particular, it may necessitate hospitalization or admission to the acute care unit, or it may lead to intubation and, in severe cases, death [23].

Thrombosis, as well as a tendency to develop thrombosis, may accompany a viral infection [24], and it has been reported to have a relationship with COVID-19 infection [25,26]. In particular, COVID-19 has been proposed to be a thrombogenic virus [27,28] and causes thromboembolic disease and cardiac thrombotic complications [29]. In accordance, microthrombi have been observed at autopsy in the lungs of patients who have died from the SARS-CoV-2 infection [30]. Thrombotic markers, such as fibrinogen and d-dimers, have been shown to increase during COVID-19 infection [31,32]. D-dimers may be a predictive factor in identifying thrombosis, and high levels may be an index of the severity of the infection and death [27]. Anticoagulation is necessary, and heparin is considered the preferred anticoagulant as it may have antiviral action [27]. In the paper herein, we measured d-dimer and fibrinogen levels and found elevated d-dimer and fibrinogen levels, which were inversely related to vitamin D levels. In accordance, low vitamin D levels have been associated with thromboembolic events [33], and vitamin D may have antithrombotic action [34].

Ferritin ensures the availability of iron for cellular metabolism, and it may be protective of the DNA and proteins from any toxic effects of iron [35]. Elevated levels of ferritin are observed in infections [36] and are thought to be an inflammatory index; it appears to be an important host defense mechanism against bacteria as it acts to limit the availability of iron, which is necessary for bacterial metabolism in the context of nutritional immunity [37]. Vitamin D has been shown to be implicated in iron metabolism and decreases hepcidin levels [38]. Ferritin levels were shown to increase during the course of SARS-CoV-2 infection, and the role of ferritin in the disease course has been previously discussed [32,39]. In particular, increased ferritin levels may be an index of severity, as well as a prognostic factor in SARS-CoV-2 patients [40]. In our study, we observed high ferritin levels in patients with COVID-19 disease, which were inversely related to vitamin D levels.

The aim was to describe the association of vitamin D levels with disease outcomes in a cohort of patients hospitalized for severe SARS-CoV-2 infection. Low vitamin D levels were observed in patients with severe SARS-CoV-2 infection, which were related to the outcome of the infection. Vitamin D levels were inversely related to d-dimer, fibrinogen, and ferritin levels.

## 2. Materials and Methods

In a group of 88 patients, 47 males and 41 females, hospitalized in the COVID-19 Department over a period of 12 months, C-reactive protein (CRP) levels, ESR levels, hemoglobin levels, white blood cell count, ferritin levels, fibrinogen levels, prothrombin levels, d-dimer levels, and 25(OH)D_3_ levels were measured (Table 1). 25(OH)D_3_ levels were estimated upon admission to the COVID-19 unit. The levels of 25(OH)D_3_ were also measured in a cohort of 88 control subjects matched for age and sex. The study was a 12-month prospective study. Patients receiving treatment with calcium and vitamin D supplements were excluded from the study. Patients receiving treatment with antihypertensives, which could have affected vitamin D levels, were excluded from the study. All patients were hospitalized with severe disease and pneumonia. None of them were asymptomatic. None of the patients were receiving treatment with drugs such as antiepileptics, long-term corticosteroids, antineoplastic drugs, or antibiotics, which could have affected vitamin D levels. Patients with comorbidities that could have affected vitamin D levels were excluded from the study.

25(OH)D_3_ was assessed by a 1-step delayed chemiluminescent microparticle immunoassay (Abbott Park, IL, USA) [41]. It is a fully automated immunoassay offered by Abbott on the ARCHITECT platform with a within-run CV of 3.0–5.4% and a between-run CV of 4.7–6.3%.

CRP was analyzed by particle-enhanced immunonephelometry (CardioPhase hsCRP; Siemens Healthcare Diagnostics, Camberley, UK). The method was based on the aggregation of polystyrene particles coated with monoclonal antibodies specific to human CRP when mixed with samples containing CRP. The equipment applied was provided by Elite Medical, Marietta, GA, USA. The formed aggregates scattered a beam of light that passed through the sample. The intensity of the scattered light was proportional to the concentration of CRP in the sample. The result was estimated as compared with a standard of known concentrations. The sensitivity of the assay was 0.175 mg/L with a coefficient of variation of 7.6% at 0.41 mg/without any known cross-reactivity. The intra-assay and inter-assay coefficients of variation ranged from 2.7% at 14 mg/L to 4.6% at 5.95 mg/L and from 2.0% at 14 mg/L to 4.0% at 5.95 mg/L, respectively.

TSH was estimated in serum by the ARCHITECT TSH immunoassay (Abbott Park, IL, USA). The latter is a chemiluminescent microparticle immunoassay. The method is characterized by an analytical sensitivity of <0.0025 μIU/mL, a precision of <10%, and an inter-assay coefficient of variation of <20%. The ARCHITECT TSH assay is a two-step immunoassay that uses chemiluminescent microparticle immunoassay technology with flexible assay protocols, known as Chemiflex. In the first step, the sample, anti-β TSH antibody-coated paramagnetic microparticles, and TSH Assay Diluent were combined. TSH present in the sample was bound to the anti-TSH antibody-coated microparticles. After washing, anti-α TSH acridinium-labeled conjugate was added in the second step. After the addition of Pre-Trigger and Trigger Solutions to the mixture, the chemiluminescent reaction was estimated as relative light units. The measurement was based on the direct relationship between the amount of TSH in the sample and the relative light units detected.

Free T_3_ (FT_3_) levels were analyzed using the ARCHITECT FT_3_ assay, which is a chemiluminescent microparticle immunoassay (Abbott Park, IL, USA) with an analytical sensitivity of <1.0 pg/mL and an analytical specificity of <0.001%. The ARCHITECT Free T_3_ assay is a two-step immunoassay for the determination of free T_3_ in human serum and plasma using Chemiluminescent Microparticle Immunoassay (CMIA) technology. In the first step, the sample and anti-T_3_-coated paramagnetic microparticles were combined. Free T_3_ (unbound) in the sample bonded to the anti-T_3_-coated microparticles. After washing, T_3_ acridinium-labeled conjugate was added. Pre-Trigger and Trigger Solutions were then added to the reaction mixture; the resulting chemiluminescent reaction was measured as relative light units. An inverse relationship existed between the amount of FT_3_ in the sample and the relative light units detected by the ARCHITECT optical system.

Free T_4_ (FT_4_) levels were estimated by the ARCHITECT FT_4_ assay, which is a chemiluminescent microparticle immunoassay (Abbott Park, IL, USA). The method possesses an analytical sensitivity of <0.4 ng/dL and a precision of <10%. The ARCHITECT Free T_4_ assay is a two-step immunoassay, which utilizes Chemiluminescent Microparticle Immunoassay (CMIA) technology. In the first step, the sample and anti-T_4_-coated paramagnetic microparticles were combined. FT_4_ (unbound) present in the sample bonded to the anti-T_4_-coated microparticles. After washing, the T_4_ acridinium-labeled conjugate was added in the second step. Pre-Trigger and Trigger Solutions were then added to the reaction mixture; the resulting chemiluminescent reaction was measured as relative light units. An

Ferritin levels were analyzed by the Atellica IM Fer assay (Siemens Healthlineers, Tarrytown, NY, USA), inverse relationship exists between the amount of Free T_4_ in the sample and the relative light units, which is a 2-site sandwich immunoassay using direct chemiluminometric technology, which utilizes constant amounts of 2 anti-ferritin antibodies. The method is characterized by an analytical sensitivity of ≤0.5 ng/mL and a limit of detection of ≤1.0 ng/mL. The Atellica IM Fer Assay utilizes 2 anti-ferritin antibodies; the first antibody is a goat polyclonal anti-ferritin antibody labeled with acridium ester and the second antibody is a mouse monoclonal anti-ferritin antibody, which is covalently coupled to paramagnetic particles. The measurement of ferritin was based on the relationship between the amount of ferritin in the sample and the amount of relative light units detected.

D-dimer levels were studied by the Innovance D-Dimer immunoturbidimetric assay (Siemes Healthlineers) with a sensitivity of <0.5 mg/L and a precision CV% between 5.9 and 8.4%. The age-adjusted sensitivity of the Innovance D-Dimer immunoturbidometric assay was 98.9% (CI 95%) and the age-adjusted specificity was 77.4% (CI 95%).

Fibrinogen levels were determined using the Multifibren U assay (Siemens Healthineers), which uses a modification of the Clauss method, with a precision within-series CV% of 2.9–7.2%. The Clauss fibrinogen assay is a quantitative, clot-based, functional assay. The assay measures the ability of fibrinogen to form a fibrin clot after being exposed to a high concentration of purified thrombin. Multifibren U is an in vitro diagnostic reagent for the quantitative determination of fibrinogen in human sodium-citrated plasma by means of automated and manual coagulometric methods. Fibrinogen determination by Multifibren U was standardized against the reference methods by Ratnoff, Menzie, and Kjeldahl [42]. Multifibren U utilizes a modification of the Clauss method. Citrated plasma was brought to coagulation by a large excess of thrombin. The coagulation time was largely dependent on the fibrinogen content of the specimen.

For the evaluation of the results of the present study on 25(OH)D_3_ as far as disease outcome (survival versus non-survival) was concerned, the levels of 25(OH)D_3_ were classified as deficiency (0–10 ng/mL), insufficiency (10–20 ng/mL), and sufficiency > 20 ng/mL.

The study was approved by the ethical committee of Asclepeion Hospital (approval number 15335, 24 November 2020). Informed consent was obtained from the patients involved in the study.

Statistical evaluation of the data was performed using the SPSS statistical package (IBM SPSS Statistics v27). Data were shown as the mean ± SD. Student’s *t*-test was used to compare the patient group with the control group. Regression analysis was performed to analyze the relationship between 25(OH)D_3_, CRP, ferritin, d-dimer, and fibrinogen levels. Statistical significance was set at a *p*-value of <0.05.

## 3. Results

**Figure 1:**
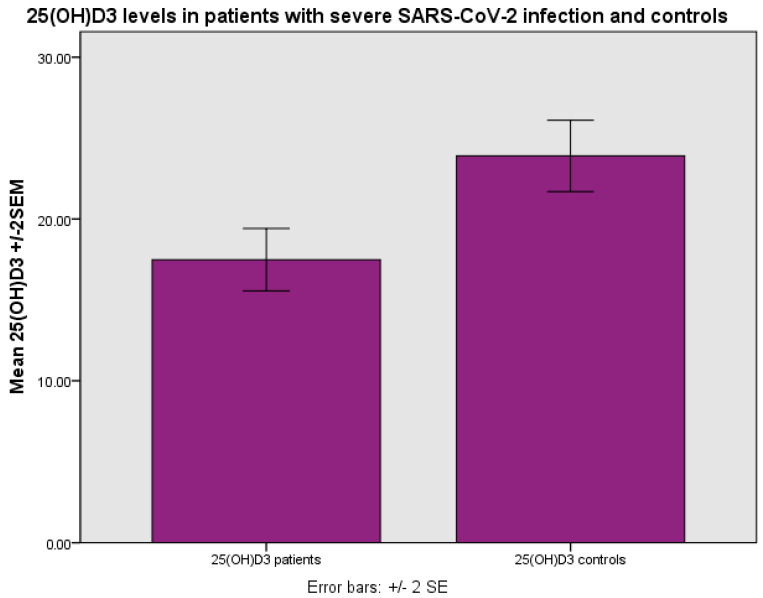
Levels of 25(OH)D_3_ (ng/mL) in patients with severe SARS-CoV-2 infection and controls, *p* < 0.001 (Student’s test).

**Figure 2:**
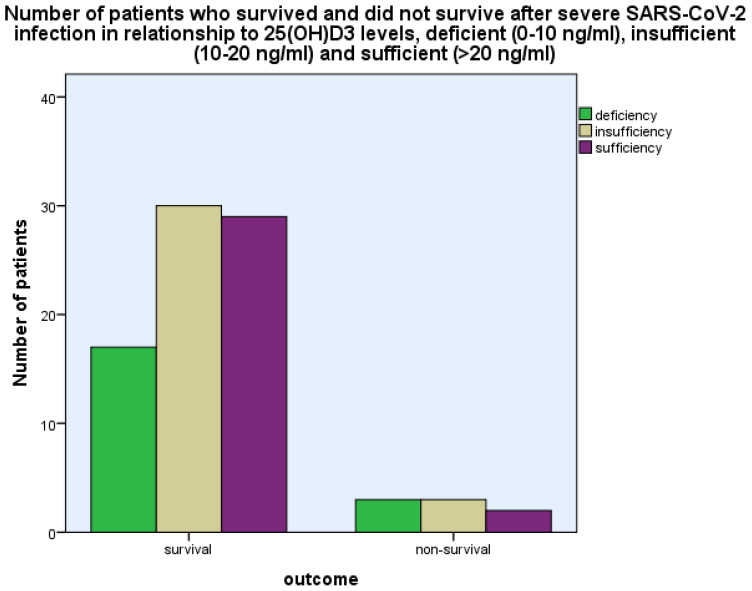
The number of patients who survived and did not survive after severe SARS-CoV-2 infection in relation to 25(OH)D_3_ concentration, deficient (0–10 ng/mL), insufficient (10–20 ng/mL) and sufficient (>20 ng/mL), *p* < 0.001, chi-square test, *p* < 0.001, Fisher’s exact test.

The levels of 25(OH)D_3_ were 17.36 ± 8.80 ng/mL (mean ± SD) as compared with 24.34 ± 10.34 ng/mL in the control group, *p* < 0.001 (Student’s *t*-test) (Figure 1). The levels of 25(OH)D_3_ were related to the outcome, i.e., survival as opposed to non-survival, as more patients with 25(OH)D_3_ deficiency (0–10 ng/mL) and insufficiency (10–20 ng/mL) had a fatal outcome as compared with those with 25(OH)D_3_ sufficiency (>20 ng/mL) (*p* < 0.001, chi-square test, *p* < 0.001, Fisher’s exact test (Figure 2)).

**Figure 3:**
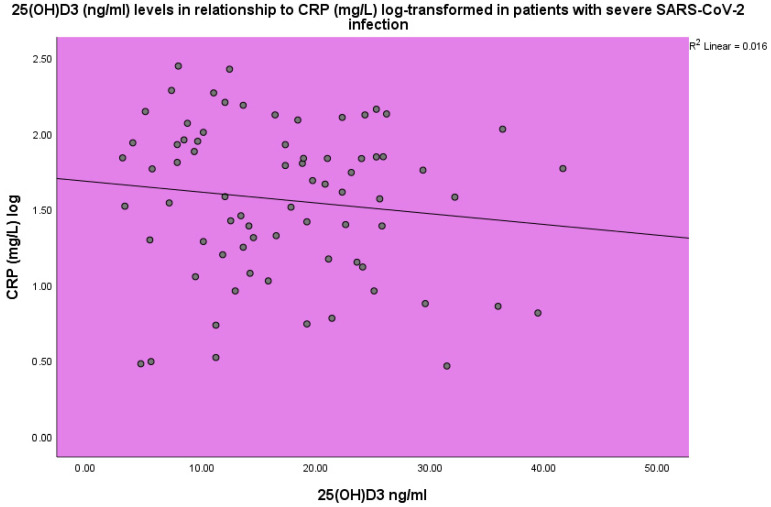
25(OH)D_3_ (ng/mL) levels in relation to CRP (mg/L) in severe SARS-CoV-2 infection, linear regression analysis, *p* < 0.001. Circles represent patient values and the line represents the regression line.

Increased CRP and ESR levels were observed in the COVID-19 patients. The concentration of 25(OH)D_3_ was found to be inversely related to CRP (*p* < 0.001, linear regression analysis, standardized coefficient of variation beta −0.176 (Figure 3)).

**Figure 4:**
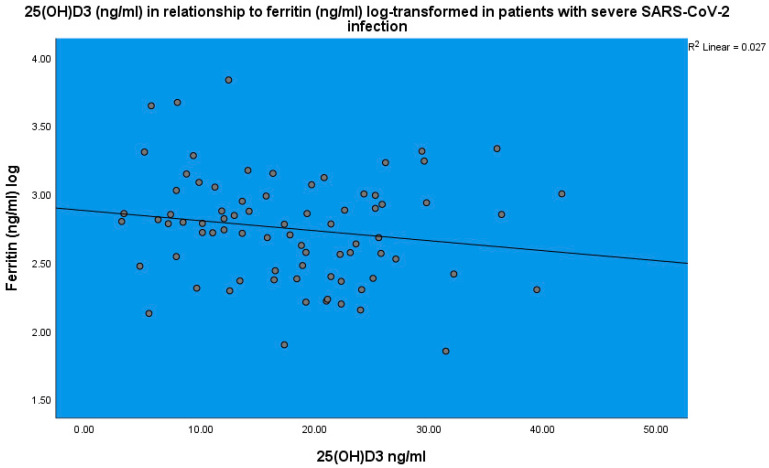
25(OH)D_3_ (ng/mL) levels in relation to ferritin concentration (ng/mL) in patients with severe SARS-CoV-2 infection, linear regression analysis, *p* < 0.001. Circles represent patient values and the line represents the regression line.

**Figure 5:**
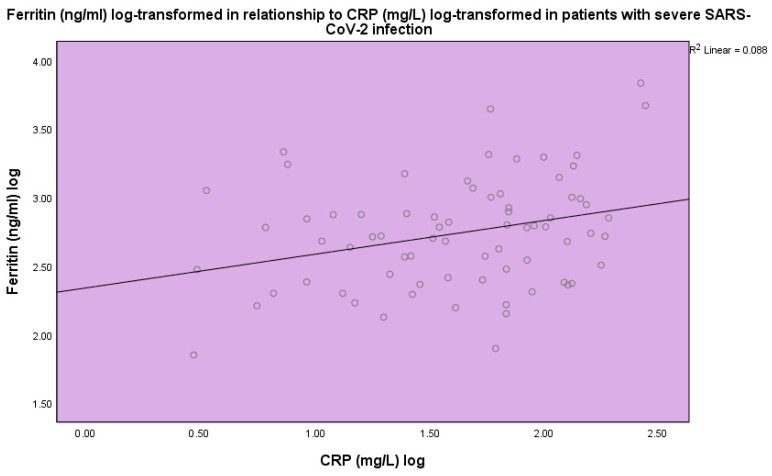
Levels of ferritin (ng/mL) in relation to CRP (mg/L) in patients with severe SARS-CoV-2 infection, linear regression analysis, *p* < 0.001. Circles represent patient values and the line the regression line.

25(OH)D_3_ was inversely related to ferritin (*p* < 0.001, linear regression analysis, standardized coefficient of variation beta −0.160 (Figure 4)). Ferritin (ng/mL) levels were found to be related to CRP (*p* < 0.001, linear regression analysis, standardized coefficient of variation beta 0.484 (Figure 5)).

**Figure 6:**
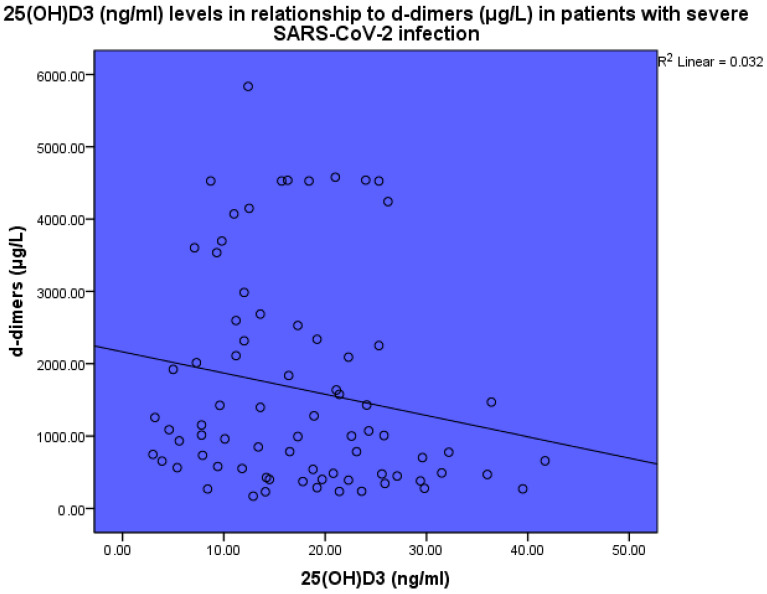
The levels of 25(OH)D_3_ (ng/mL) in relation to d-dimers (μg/L) in patients with severe SARS-CoV-2 infection, linear regression analysis, *p* < 0.001. Circles represent patient values and the line represents the regression line.

**Figure 7:**
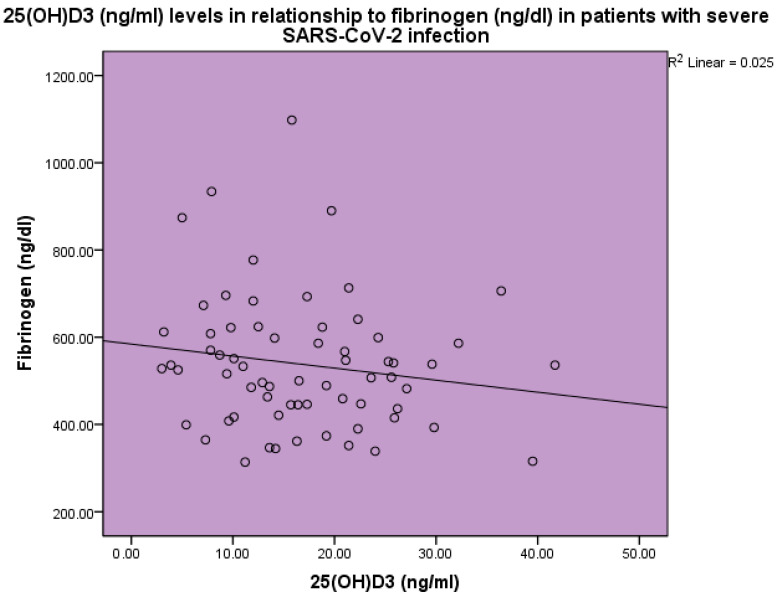
The levels of 25(OH)D_3_ (ng/mL) in relation to fibrinogen (ng/dL) in patients with severe SARS-CoV-2 infection, linear regression analysis, *p* < 0.001. Circles represent patient values and the line represents the regression line.

Thrombotic markers were found to be increased in the blood of sick patients as compared to the expected normal levels. 25(OH)D_3_ levels were inversely related to d-dimer levels (*p* < 0.001, linear regression analysis, standardized coefficient of variation beta −0.178 (Figure 6)). 25(OH)D_3_ levels were inversely related to fibrinogen levels (*p* < 0.001, linear regression analysis, standardized coefficient of variation beta −0.158 (Figure 7)).

## 4. Discussion

SARS-CoV-2 infection may lead to a severe acute disease that necessitates hospitalization and may lead to intubation and death [43,44]. Various agents have been employed to combat the SARS-CoV-2 infection, amongst which are vaccines and antiviral medications. As technology was quick to employ the proper vaccines to combat the COVID-19 pandemic [45,46], antiviral medications were subsequently discovered [47,48,49] and the use of various agents that might aid in fighting the disease gained the attention of the scientific community. Dexamethasone was found to improve the course of the disease and outcomes. Vitamin D, when insufficient, may lead to a worse outcome, whereas vitamin D administration may improve this outcome [17,18,19]. In the present study, vitamin D insufficiency was observed in SARS-CoV-2 patients and was related to disease outcome.

Vitamin D deficiency has been described in severe SARS-C0V-2 infections [4,50,51,52,53,54,55,56,57]. Bassatne et al. [51], in a meta-analysis of 31 peer-reviewed observational studies, found a positive trend between levels of 25(OH)D3 < 20 ng/mL and an increased risk of mortality, ICU admission, and mechanical ventilation, both invasive and non-invasive. Chiodini et al. [4] performed a meta-analysis of 54 studies on SARS-CoV-2 infection and vitamin D and found that severe deficiency, deficiency, and insufficiency of vitamin D was related to hospitalization, the need for admission to the intensive care unit, mortality, and COVID-19 infection. They concluded that individuals with vitamin D deficiency present with an increased risk of the development of acute respiratory distress syndrome, requiring admission to the intensive care unit, higher mortality, and an increased susceptibility to SARS-CoV-2 infection and hospitalization. Contreras-Bolivar et al. [58] reviewed the association between vitamin D and COVID-19 and concluded that there are biological data linking vitamin D to the cytokine storm, which may lead to the severe consequences of the SARS-CoV-2 infection, like the acute respiratory distress syndrome. They also concluded that vitamin D supplementation might be a useful strategy for the prevention of COVID-19 disease in vitamin D-deficient populations. Mohan et al. [50] reviewed the association between vitamin D and COVID-19 disease and concluded that vitamin D may stop the hyperinflammatory reaction to the virus and may speed up the healing process in the lungs. In our study, we observed low vitamin D levels in severe SARS-CoV-2 infection and a relationship between vitamin D deficiency and insufficiency with outcomes, i.e., a fatal outcome as opposed to survival. Vitamin D is an immunomodulatory hormone that acts in a pleiotropic way by both enhancing innate immunity and the response of the organism against various infections and suppressing the hyperinflammatory response to the viral insult [59]. Vitamin D has been shown to contribute to the response to various pathogens [11,60], especially those attacking the respiratory system [61]. Thus, vitamin D may enhance the innate immune response against the virus and may dampen the hyperinflammatory reaction against the COVID-19 virus [59]. Vitamin D enhances the innate immune response against the SARS-CoV-2 virus by activating toll-like receptor 2 and via peptide synthesis, which acts to combat infectious invasions [62]. Vitamin D decreases proinflammatory cytokine release via CD4+ lymphocytes, thus inhibiting the development of a cytokine storm [63]. Additionally, vitamin D increases the bioavailability and expression of ACE2 [64], which acts as a receptor for the virus and may contribute to the trapping and inactivation of the virus [65]. However, it has been discussed that vitamin D deficiency observed in patients with SARS-CoV-2 infection might be the result of reverse causation, i.e., the inflammatory process leads to low vitamin D levels, as vitamin D may be a negative acute phase reactant [66,67]. Additionally, corticosteroid administration for the treatment of COVID-19 disease may have contributed to the detection of low vitamin D levels [68]. Vitamin D binding protein (DBP) is a transporter and reservoir of vitamin D metabolites [69]. Approximately 85% of vitamin D is bound to DBP, another 12% approximately is bound to albumin, and the remaining fraction circulates in a free form. In humans, there are significant DBP polymorphisms, among which the genetic polymorphisms rs7041 and rs 4588 affect the plasma concentrations of 25(OH)D and 1,25(OH)2D [70]. It has been suggested that DBP polymorphisms may affect vitamin D metabolism and bioavailability and COVID-19 infection and its prognosis [71].

Vitamin D supplementation may play a significant role in the protection of the human organism against COVID-19 infection. The effect of vitamin D supplementation on the prognosis of COVID-19 infection has been systematically investigated. In particular, in the context of the COVIT-TRIAL, a randomized controlled study, the effect of vitamin D supplementation on the prognosis of COVID-19 infection in high-risk older patients was assessed [72]. In the latter [73], which was a multicenter, randomized, controlled, open-label study and took place in France, patients were randomly allocated to either single oral high-dose (400,000 IU) or standard-dose (50,000 IU) cholecalciferol administered after COVID-19 infection. The primary outcome was 14-day mortality from any cause. A group of 254 patients with a median age of 88 years met the eligibility criteria and formed the intention-to-treat group. On the whole, 8 (6%) of 127 patients allocated to high-dose cholecalciferol and 14 (11%) of 127 patients allocated to standard-dose cholecalciferol died within 14 days, *p* = 0.049. However, the protective effect of the oral cholecalciferol administration was not sustained for up to 28 days, although also did not lead to more frequent adverse effects as compared to the standard vitamin D dose. Bilezikian et al. [74] suggested that vitamin D administration should be individualized according to the needs of the patients and the existing comorbidities, such as kidney and liver disease, which may affect vitamin D metabolism.

Vitamin D levels were found to be inversely related to CRP. This finding has been previously observed [75] in patients with autoimmunity and vitamin D deficiency [76] and in patients hospitalized in the acute care unit [75]. Thus, vitamin D may be a reverse acute-phase reactant or a reverse index of the acute-phase response [77]. Ferritin levels were found to have increased as opposed to the expected normal range in patients with severe SARS-CoV-2 disease in this study, and a negative association was observed between vitamin D and ferritin levels. Ferritin was increased in patients with COVID-19 disease and its levels were found to be related to disease severity and disease outcome. In a meta-analysis involving 52 studies, the authors [39] observed higher ferritin levels in patients with severe COVID-19 infection in comparison to patients with mild infection. The SARS-CoV-2 infection has been included in hyperferritinemic syndromes [78]. Hyperferritinemia in critically ill COVID-19 patients may be an index of severity as well as a pathogenic mediator [79]. Ferritin in the context of severe COVID-19 disease may act as a possible enhancer of the cytokine storm and may be involved in a vicious pathogenic loop [40,80,81]. In a retrospective cohort study, the association between ferritin levels, inflammatory markers, and the prognosis of the SARS-CoV-2 infection was investigated. In particular, the association between ferritin, soluble CD163, IL-18, and COVID-19 prognosis was assessed [81]. Clinical and laboratory parameters of 70 patients with severe infection from the SARS-CoV-2 virus were analyzed. Ferritin, CD163, and IL-18 were assayed. The aim of the study was to evaluate the relationship between the above-mentioned parameters and respiratory outcomes and overall survival. A group of 60 patients survived 30 days of hospitalization. Significant differences were observed between subjects who were alive following 30 days in comparison to those who did not survive. Higher levels of IL-18 and ferritin were observed in the group with fatal outcomes as compared to those who survived. Soluble CD163, ferritin, and IL-18 levels were correlated with COVID-19 disease severity. It was suggested that the measurement of ferritin and IL-18 during the disease may help in the refinement of the decision-making process related to the necessity for hospitalization [81]. As found in our study, a relationship was observed between CRP and ferritin as both increased during an infection.

Viral infections are related to a tendency to develop thrombosis [82]. The SARS-CoV-2 infection is related to the development of both arterial and venous thrombosis [83]. Savla et al. [84] discussed the coagulation complications of COVID-19 infection and their relationship with the cytokine storm. In an autopsy study of pulmonary tissue obtained from patients who succumbed to COVID-19 disease, histologic analysis revealed thrombosis and microangiopathy [30]. Alveolar capillary microthrombi were prevalent in patients who died from COVID-19. In COVID-19 infection viral effects, increased vasoconstrictor angiotensin II and decreased vasodilator angiotensin and cytokine release in the context of sepsis may lead to coagulopathy. Coagulopathy may be observed in as many as 50% of patients with severe COVID-19 disease.

Studies have shown increased d-dimer and fibrinogen levels in COVID-19 disease during the course of the illness [85]. A 3- to 4-fold rise in d-dimer levels may be related to poor prognosis [85]. A meta-analysis of 29 studies [86] showed that in patients with SARS-CoV-2 disease, increased d-dimers on admission were related to an augmented risk of disease severity and mortality. In our study, increased levels of d-dimer and fibrinogen were observed in severe SARS-CoV-2 infection as compared with healthy controls. D-dimer and fibrinogen levels were inversely related to 25(OH)D_3_ levels. In a small study, short-term high-dose vitamin D supplementation induced a significant decrease in fibrinogen concentrations, without, however, an effect on CRP, d-dimer, or ferritin levels, leading to the conclusion that vitamin D might have an antithrombotic effect [87]. Additionally, vitamin D has been shown to possess in vitro antithrombotic effects via the inhibition of IL-6 [88].

In conclusion, lower vitamin D levels were observed in patients with severe SARS-CoV-2 disease as compared to control subjects. Vitamin D deficiency and insufficiency were related to disease outcomes. Vitamin D levels were inversely related to CRP, ferritin, d-dimer, and fibrinogen levels.

## 5. Conclusions

In conclusion, low vitamin D levels were observed in patients with severe SARS-CoV-2 infection, which were related to the disease outcome and inversely related to ferritin, d-dimer, and fibrinogen levels.

## Figures and Tables

**Table 1:** Characteristics of patients hospitalized for severe SARS-CoV-2 infection.

	Normal Range	Minimum	Maximum	Mean	Std. Deviation
Age (years)		21.0	97.0	67.7	16.9
Sex (male/female)	47/41				
White blood cell count (cells/μL)	4.6–10.2	0.71	17,300.0	6259.6	3373.5
Neutrophils (%)		37.6	89.9	70.5	13.1
CRP (mg/L)	<5.0	2.9	280.0	65.1	60.6
ESR (mm/1 h)	<15	15.0	108.0	44.6	24.4
25(OH)D_3_ (ng/mL)		3.0	41.7	17.4	8.8
Ferritin (ng/mL)	20–300	71.0	6821.0	851.9	1032.6
d-dimers (μg/L)	0–550	170.0	5835.0	1690.9	1479.5
Fibrinogen (ng/dL)	180–350	314.0	1098.0	543.1	157.2
TSH (μIU/mL)	0.35–4.94	0.36	3.36	1.17	0.78
FT_3_ (pg/mL)	1.58–3.91	2.2	5.1	3.7	0.66
K (mmol/L)	3.5–5.2	2.2	5.1	3.7	0.6
PT (s)		10.0	14.3	11.7	1.0
APTT (s)	26–39	20.9	59.3	31.1	5.1
INR		0.86	1.29		0.1

## Data Availability

Data are available at the database of Asclepeion Hospital, Voula, Athens, Greece.
